# First Experience of Single Port Robotic Areolar (SPRA) Thyroidectomy and Modified Radical Neck Dissection (MRND)

**DOI:** 10.3390/medicina61071150

**Published:** 2025-06-25

**Authors:** Myung Ho Shin, Yue Kun Yin, Hilal Hwang, Sun Min Lee, Jin Wook Yi

**Affiliations:** 1 Department of Surgery, Inha University Hospital, Inha University College of Medicine, Incheon 22332, Republic of Korea; 2 Thyroid Cancer Center, AIN Hospital, Incheon 22148, Republic of Korea

**Keywords:** thyroid, modified radical neck dissection, robotic surgical procedures, minimally invasive surgical procedures

## Abstract

*Background and Objectives*: After introducing the single-port robotic surgical system (da-Vinci SP), thyroid surgeries using da-Vinci SP are becoming more popular. Although many methods have been designed for thyroidectomy using the da-Vinci SP, there are very few reports on methods that can perform not only thyroidectomy but also lateral cervical lymph node dissection. In this study, we want to report the first clinical experience with SPRA-MRND (Single Port Robotic Areolar-Modified Radical Neck dissection), using right breast access. *Materials and Methods*: From April 2024 to January 2025, a total of 24 robotic MRNDs were performed, of which 11 were SPRA-MRNDs. The remaining 13 were performed using conventional BABA surgery. The two data sets were compared through retrospective medical record analysis. *Results*: There were no significant differences regarding patient characteristics, pathologic variables and oncologic outcomes between the two groups. However, SPRA group showed significantly shorter operation time (182.1 ± 27.5 vs. 213.1 ± 31.5 min, *p* = 0.017), higher immediate postoperative calcium level (calcium: 8.7 ± 0.5 vs. 8.0 ± 0.8 mg/dL, *p* = 0.014) and lower drainage amount (98.1 ± 33.2 vs. 146.4 ± 43.2 mL, *p* = 0.005). *Conclusions*: Our initial experience has shown that SPRA-MRND is performed safely. We propose SPRA-MRND as a good method for minimally invasive robotic surgery.

## 1. Introduction

Thyroid cancer is the most common endocrine malignancy, with its incidence steadily increasing worldwide over the past decades, especially among women in their third to fifth decades of life [1,2]. The majority of thyroid cancers are papillary thyroid carcinoma (PTC), which is known for its indolent clinical course and favorable prognosis. However, PTC frequently metastasizes to regional lymph nodes, necessitating comprehensive lymph node dissection for accurate staging and oncologic control. In cases where metastatic lymph nodes are located lateral to the internal jugular vein and carotid artery, the disease is classified as N1b stage, which requires lateral neck dissection for appropriate management [3,4].

Lateral neck dissection for PTC with N1b disease is commonly performed as a modified radical neck dissection (MRND), which involves the removal of lymph nodes from levels II to V while preserving key anatomical structures such as the sternocleidomastoid muscle, internal jugular vein, and spinal accessory nerve [5,6]. MRND represents the most extensive procedure among thyroid cancer surgeries. Conventionally, it is performed via a large transverse cervical incision measuring 15 to 20 cm across the anterior neck [6]. While this approach provides direct access and visibility, the resulting prominent scar can lead to significant cosmetic and psychological distress—particularly in young female patients, who constitute the majority of thyroid cancer cases. Consequently, visible scarring can negatively impact their self-image and social interactions [6,7].

To mitigate these cosmetic concerns, various remote-access thyroidectomy techniques have been developed, initially using endoscopic methods and subsequently advanced by the integration of robotic systems. Robotic thyroid surgeries using transaxillary, bilateral axillo-breast approach (BABA), and transoral approach have been widely adopted and validated for thyroidectomy and central neck dissection [8,9]. However, reports of MRND performed via remote-access techniques remain limited due to technical complexity and limited access to lateral neck compartments. In 2021, we previously reported the feasibility of robotic MRND using the BABA method with the da Vinci Xi system, demonstrating comparable oncologic outcomes to open surgery [10]. Nonetheless, the BABA technique necessitates wide subcutaneous flap dissection, challenging its classification as a truly minimally invasive procedure.

To overcome this limitation, we developed a novel surgical method known as single-port robotic areolar thyroidectomy (SPRA), utilizing the da Vinci SP system through a single right areolar incision without axillary dissection. This technique, first reported in 2023, significantly reduces the subcutaneous flap area by over 50% compared to BABA, and completely avoids visible scarring [11]. Subsequently, we published a one-year comparative study demonstrating the clinical safety and reduced invasiveness of SPRA over BABA in more than 100 cases [12]. Recently, we successfully extended the SPRA approach to perform MRND for patients with lateral neck metastasis. To our knowledge, this is the first report worldwide of SPRA-MRND. In this study, we present the perioperative and oncologic outcomes of SPRA-MRND and compare them to the established BABA-MRND technique.

## 2. Materials and Methods

Clinical and perioperative variables were extracted from electronic medical records. These included age, sex, body mass index (BMI), side of MRND (left vs. right), postoperative hospital stay, incidence of vocal cord palsy and hypoparathyroidism, daily drain amount during admission, visual analogue scale (VAS) scores for pain (0 to 10), and serum thyroglobulin (Tg) levels—both unstimulated and TSH-stimulated—prior to radioactive iodine (RAI) ablation.

Operative details were obtained from intraoperative records and surgical video analysis. “Flap time” was defined as the duration from insertion of the robotic endoscope to completion of the subcutaneous flap before robot docking. “Console time” referred to the duration from activation of the robotic arms at the surgeon console to the completion of thyroid and neck dissection. “Total operative time” was obtained from anesthetic records.

Postoperative vocal cord function was evaluated by voice assessment and vocal cord ultrasonography. Hypoparathyroidism was defined as the presence of clinically significant hypocalcemic symptoms requiring calcium supplementation; transient and permanent cases were differentiated based on recovery status within 6 months.

Pathologic variables included tumor size, multifocality, margin status, microscopic or gross extrathyroidal extension (ETE), lymphovascular invasion, and number of lymph nodes retrieved and involved per level (levels II, III, IV, V, and VI). The presence of *BRAF*^V600E^ and *TERT* promoter mutations was also documented. Laboratory findings included postoperative serum calcium, ionized calcium, and parathyroid hormone (PTH) levels.

The indications for robotic MRND at our institution were comparable to those for open surgery, with the only absolute contraindication being evidence of T4 disease on preoperative imaging—defined as direct invasion of the trachea, esophagus, vertebra, or major vessels.

We used R programming language version 4.4.2 (R core team, Vienna, Austria). Continuous variables were compared using the unpaired *t*-test, including mean and standard deviation. Fisher’s exact test was applied for categorical variables due to the small numbers between two groups. A *p*-value of 0.05 or less was considered statistically significant.

### Surgical Technique

**Figure 1:**
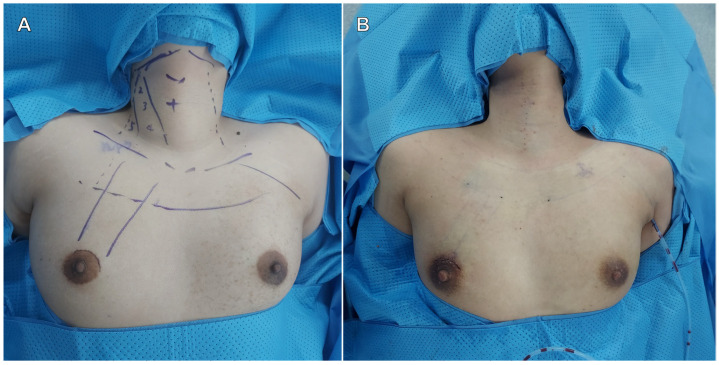
Flap design (**A**) and postoperative surgical wound (**B**).

In the SPRA group, the subcutaneous flap was designed as shown in Figure 1A, with a 3-cm supra-areolar incision used to elevate the flap, followed by docking of the da Vinci SP system. A 5-mm auxiliary trocar was inserted in the medial axilla for flap dissection, suction, and irrigation; this site was also used for placement of a Jackson-Pratt drain postoperatively (Figure 1B).

**Figure 2:**
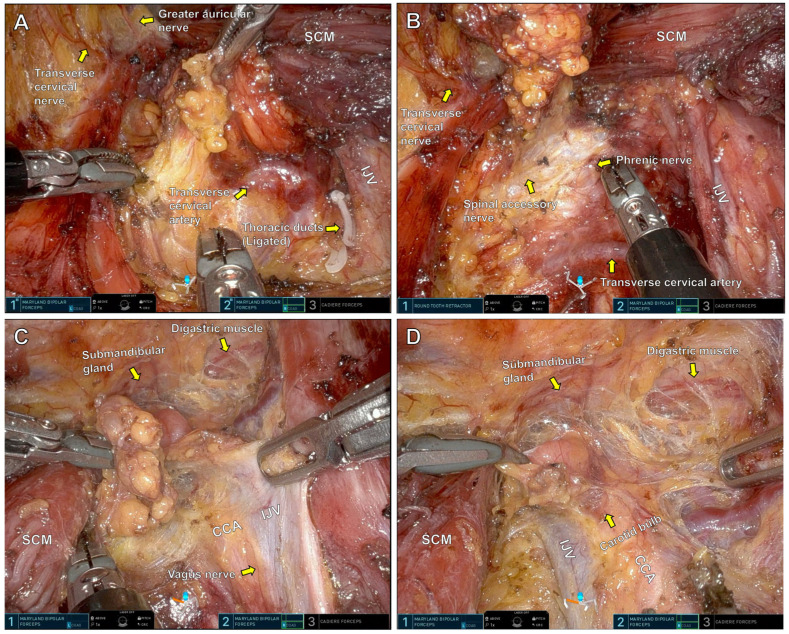
Right MRND surgical field under the da-Vinci SP console. Level 4 (**A**), level 3 (**B**), level 2B (**C**) and level 2A (**D**). SCM: Sternocleidomastoid muscle, IJV: Internal jugular vein, CCA: Common carotid artery.

Although the SPRA technique for thyroidectomy has been previously described [11], this study focused on its extension to MRND. The detailed procedural steps are illustrated in Figure 2.

Figure 2A: Lymph node dissection begins at levels 5 and 4. The transverse cervical artery is identified as a key anatomical landmark. Lymphatic tissue is dissected along the internal jugular vein. The thoracic duct, if encountered, is ligated with robotic Hem-o-lok clips to prevent chyle leakage. Careful dissection along the lateral border of the sternocleidomastoid muscle preserves sensory nerves emanating from Erb’s point.

Figure 2B: Dissection continues superiorly through level 3. The internal jugular vein is skeletonized while preserving the phrenic nerve and spinal accessory nerve.

Figure 2C: Posterolateral lymph nodes at level 2B are dissected along the bifurcation of the internal jugular vein. Dissection continues cephalad until the submandibular gland and digastric muscle are visualized. The vagus nerve, located between the internal jugular vein and common carotid artery, is carefully preserved.

Figure 2D: Lymph nodes in level 2A, anterior to the jugular bifurcation, are dissected with attention to preserving the carotid bulb and avoiding injury to the common carotid artery.

## 3. Results

A total of 24 patients underwent robotic total thyroidectomy with modified radical neck dissection (MRND) during the study period. Of these, 13 patients underwent surgery via the bilateral axillo-breast approach (BABA group), and 11 patients underwent the single-port robotic areolar approach (SPRA group).

As summarized in Table 1, there were no significant differences in baseline demographics between the BABA and SPRA groups, including age (39.4 ± 12.0 vs. 41.7 ± 11.1 years, *p* = 0.624), gender distribution (*p* = 0.098), and body mass index (23.5 ± 3.5 vs. 22.0 ± 3.1 kg/m^2^, *p* = 0.269). The side of MRND (left vs. right) did not differ significantly between groups (*p* = 0.444). Regarding operative parameters, the operation time for flap was significantly shorter in the SPRA group (17.2 ± 3.9 vs. 22.2 ± 6.0 min, *p* = 0.024), as was the total operation time (182.1 ± 27.5 vs. 213.1 ± 31.5 min, *p* = 0.017). Operation time in console was shorter in SPRA group but had no significant difference (136.8 ± 25.3 vs. 148.2 ± 31.6 min, *p* = 0.339). The length of postoperative hospital stay was not different between the groups (4.4 ± 1.4 vs. 4.3 ± 0.9 days, *p* = 0.911).

Table 2 presents the postoperative outcomes. There were no significant differences in the incidence of transient or permanent vocal cord palsy (*p* > 0.4) or hypoparathyroidism (*p* = 1.000). Early postoperative calcium and ionized calcium levels (within 2 weeks) were significantly higher in the SPRA group (calcium: 8.7 ± 0.5 vs. 8.0 ± 0.8 mg/dL, *p* = 0.014; ionized calcium: 1.1 ± 0.1 vs. 1.0 ± 0.1 mmol/L, *p* = 0.012). There were no significant differences in PTH levels or calcium values beyond 6 months postoperatively. The drain amount on the first postoperative day was significantly lower in the SPRA group (98.1 ± 33.2 vs. 146.4 ± 43.2 mL, *p* = 0.005), while second-day drainage volumes were similar (62.5 ± 17.6 vs. 66.6 ± 37.2 mL, *p* = 0.724). Pain scores on postoperative days 1 and 2 were slightly lower in the SPRA group, but the differences were not statistically significant (Day 1 VAS: 2.6 ± 0.7 vs. 2.9 ± 0.3, *p* = 0.210; Day 2 VAS: 2.5 ± 0.7 vs. 2.5 ± 0.7, *p* = 0.765).

As shown in Table 3, there were no statistically significant differences in pathological variables between groups. Tumor size (1.5 ± 0.4 vs. 1.4 ± 1.1 cm, *p* = 0.943), multifocality (*p* = 1.000), extrathyroidal extension (*p* = 1.000), margin status (*p* = 1.000), lymphatic invasion (*p* = 0.232), and microvascular invasion (*p* = 0.397) were comparable. The number of metastatic lymph nodes was similar (7.6 ± 5.6 vs. 6.5 ± 4.1, *p* = 0.572), as were the total retrieved lymph nodes (25.3 ± 9.3 vs. 25.9 ± 10.7, *p* = 0.875). No differences were observed in lymph node yield across individual neck levels II through VI (all *p* > 0.4). The frequency of *BRAF*^V600E^ mutation was not significantly different between the two groups (*p* = 0.649). There was no observed *TERT* promotor mutation in both groups.

Table 4 compares postoperative thyroglobulin and RAI-related markers. There were no significant differences between groups in serum unstimulated thyroglobulin levels measured 3 months after surgery (0.3 ± 0.2 vs. 0.7 ± 1.4 ng/mL, *p* = 0.312), or in TSH-stimulated thyroglobulin levels prior to RAI administration (5.6 ± 8.6 vs. 3.5 ± 4.3 ng/mL, *p* = 0.530). The first administered RAI dose and TSH level prior to RAI also showed no significant differences between groups.

## 4. Discussion

Although the incidence of thyroid cancer has been very high recently, most cases are papillary thyroid cancer, which has a good prognosis [13]. The widespread use of high-resolution ultrasonography has contributed to increased detection of subcentimeter papillary microcarcinomas, further improving prognosis [14]. However, despite its indolence, PTC demonstrates a high propensity for cervical lymph node metastasis, with up to 30% of patients exhibiting cervical lymph node involvement [15,16]. When the lymph node metastasis extends beyond the central to the lateral neck involving the internal jugular vein and carotid sheath, it is classified as N1b disease under the AJCC staging system, associated with increased recurrence risk and a strong indication for therapeutic modified radical neck dissection (MRND), followed by radioactive iodine (RAI) ablation [3,15,17].

Surgical resection remains the cornerstone of treatment for PTC. Even in patients with distant metastasis, thyroidectomy combined with lymph node dissection is required to reduce disease burden and enhance the efficacy of adjuvant RAI therapy. For PTC without radiologically evident nodal involvement, prophylactic central lymph node dissection is often recommended, whereas therapeutic central or lateral lymph node dissection is necessary when metastatic lymph nodes are clinically or cytologically confirmed [3,4]. Central neck dissection, while associated with risk to the parathyroid glands and recurrent laryngeal nerve, typically requires only a 4–5 cm anterior neck incision. In contrast, lateral neck dissection involves resection of nodal levels II to V, requiring exposure of critical anatomical structures including the carotid artery, internal jugular vein, transverse cervical artery, vagus nerve, and thoracic duct. This is typically accomplished via a 15–20-cm low cervical incision, which often results in prominent, permanent scarring. In patients prone to hypertrophic scarring or keloids, the aesthetic and psychological burden of such incisions can be considerable [7]. Given the predominance of thyroid cancer in younger women, these postoperative sequelae can significantly impact quality of life and social activity.

To overcome the aesthetic drawbacks of conventional open surgery, various remote-access approaches have been developed, including transaxillary, bilateral axillo-breast (BABA), transoral, and retroauricular methods [9]. Initially performed using endoscopic instruments, these approaches were later refined with the introduction of the da Vinci robotic system in the mid-2000s. Despite the advantages of scarless outcomes, these techniques are often categorized as “remote access surgery” rather than “minimally invasive surgery,” since they require a wider subcutaneous flap dissection compared to the small 4–5 cm incision used in open thyroidectomy [8,9,18]. Consequently, robotic thyroidectomy has been more widely adopted in regions with higher cosmetic sensitivity, such as South Korea or Thailand, whereas its acceptance in Western countries has been limited [16,19]. However, MRND is an ideal indication for remote access surgery, as the conventional open approach is inherently more invasive and disfiguring, making robotic MRND an attractive and feasible alternative as robotic platforms become more widely available [20].

Robotic MRND has primarily been performed using multiport platforms such as the da Vinci Xi, through either transaxillary or BABA approaches [21,22]. The transaxillary route enables facile ipsilateral MRND but limits contralateral access, necessitating redocking for bilateral dissections. Recently, single-port robotic transaxillary procedures have emerged; however, bilateral MRND remains technically challenging [23,24]. The BABA method allows for bilateral MRND without redocking, but requires extensive subcutaneous flap elevation [25,26]. We previously reported the outcomes of robotic MRND via the BABA technique, demonstrating comparable oncologic outcomes and complication rates relative to open surgery [10]. Subsequently, we pioneered the SPRA technique using the da Vinci SP system, which utilizes a single right areolar incision to perform thyroidectomy with significantly reduced flap area, while preserving bilateral working space. Our prior study demonstrated the safety and efficacy of SPRA compared to BABA in over 100 patients [12]. The present study is the first to report the feasibility of MRND via SPRA, confirming its successful application in patients with lateral neck metastasis.

Our findings highlight several advantages of SPRA-MRND over other robot surgery methods. First, the SPRA approach enables bilateral thyroidectomy and lateral neck dissection without redocking. The SPRA method use only a single right areolar port, with easy access to both thyroid glands and bilateral lateral neck lymph nodes from level 2 to 5. In contrast, the transaxillary approach requires contralateral docking for bilateral lateral lymph node dissection. Therefore, the transaxillary method has the disadvantage of requiring the robot to be re-docked through the opposite axilla to perform the bilateral MRND. Second, the subcutaneous flap area in SPRA is smaller than in BABA method, resulting in significantly shorter flap dissection time (17.18 ± 3.95 vs. 22.15 ± 5.96 min, *p* = 0.024) and overall operative time (182.09 ± 27.46 vs. 213.08 ± 31.46 min, *p* = 0.017), as shown in Table 1. Reduced flap dissection may also account for the significantly lower drain output on postoperative day one (98.09 ± 33.23 vs. 146.39 ± 43.21 mL, *p* = 0.005), as shown in Table 2. This finding suggests that SPRA-MRND is literally minimally invasive compared to BABA-MRND, so surgeons can explain to patients that this method not only benefits in hiding scars but is also minimally invasive. Third, there were no differences in postoperative complications. Serum calcium and ionized calcium levels within 2 weeks were significantly higher in the SPRA group (8.69 ± 0.53 vs. 7.98 ± 0.77 mg/dL, *p* = 0.014; 1.10 ± 0.09 vs. 1.01 ± 0.08 mmol/L, *p* = 0.012). Therefore, we suggest that SPRA-RND is less likely to cause transient hypoparathyroidism, which is a finding that can be very helpful to patients. Although this may reflect improved precision of the da Vinci SP robotic arms compared to da Vinci Xi system, further research is needed to control for confounding factors. Fourth, total and level-specific lymph node yields were comparable between groups, supporting the oncologic adequacy of SPRA-MRND. Therefore, this suggests that the lateral cervical lymph node area can be thoroughly removed using the SPRA-MRND method, not falling behind the existing BABA-MRND method. Fifth, unlike transoral endoscopic or robotic approaches (TOETVA, TORT), which carry risks of infection and mental nerve injury, and which are unable to achieve the complete MRND, the SPRA-MRND approach avoids such complications and enables safe MRND, which is not feasible with transoral techniques.

Our study has several limitations. This was a retrospective, single-center, non-randomized study with a small sample size. The procedures were all performed by a high-volume endocrine surgeon with extensive experience in robotic surgery, potentially limiting generalizability. Furthermore, adoption of SPRA is currently limited by the availability of the da Vinci SP system. Although all patients in this study underwent preoperative breast imaging to exclude underlying breast pathology, long-term follow-up of the areolar access site is warranted. In Korea, training on the SPRA method is actively being conducted online and offline for endocrine surgeons at hospitals where the da Vinci SP has been installed. These institutions are continuously presenting their initial experiences with SPRA at academic conferences. Therefore, we expect that active research on the SPRA method will be conducted through multi-institutional studies in the future.

## 5. Conclusions

This study is the first to demonstrate that MRND can be safely and effectively performed using the SPRA approach. SPRA-MRND is less invasive than previously described robotic thyroidectomy techniques, while maintaining oncologic safety and improving aesthetic outcomes.

## Figures and Tables

**Table 1:** Patients and operative characteristics.

Variable	BABA (n = 13)	SPRA (n = 11)	*p*-Value
Age (years, mean ± sd)	39.4 ± 12.0	41.7 ± 11.1	0.624
Gender			
Male	4	0	0.098
Female	9	11	
BMI (Body mass index, kg/m^2^)	23.5 ± 3.5	22.0 ± 3.1	0.269
Neck dissection side			
Left MRND *	6	7	0.444
Right MRND *	7	4	
Operation time for flap (minutes, mean ± sd)	22.2 ± 6.0	17.2 ± 3.9	0.024
Operation time in console (minutes, mean ± sd)	148.2 ± 31.6	136.8 ± 25.3	0.339
Total operation time (minutes, mean ± sd)	213.1 ± 31.5	182.1 ± 27.5	0.017
Hospital admission days after surgery (days, mean ± sd)	4.3 ± 0.9	4.4 ± 1.4	0.911

* MRND: modified radical neck dissection.

**Table 2:** Postoperative outcomes.

Variable	BABA (n = 13)	SPRA (n = 11)	*p*-Value
Vocal cord palsy			1.000
Transient (<6 months)	1	0	0.482
Permanent (>6 months)	1	0	1.000
Hypoparathyroidism			
Transient (<6 months)	3	1	1.000
Permanent (>6 months)	1	0	
PTH ^†^ (pg/mL) < 2 weeks	13.0 ± 10.3	14.6 ± 9.1	0.690
PTH ^†^ (pg/mL) > 6 months	28.3 ± 16.2	22.1 ± 10.5	0.270
Calcium < 2 weeks	8.0 ± 0.8	8.7 ± 0.5	0.014
Calcium > 6 months	9.2 ± 0.4	9.1 ± 0.6	0.736
Ionized calcium < 2 weeks	1.0 ± 0.1	1.1 ± 0.1	0.012
Ionized calcium > 6 months	1.2 ± 0.1	1.2 ± 0.1	0.293
Drain amount for postoperative 1st day (mL)	146.4 ± 43.2	98.1 ± 33.2	0.005
Drain amount for postoperative 2nd day (mL)	66.6 ± 37.2	62.5 ± 17.6	0.724
VAS ^‡^ for postoperative 1st day	2.9 ± 0.3	2.6 ± 0.7	0.210
VAS ^‡^ for postoperative 2nd day	2.5 ± 0.7	2.5 ± 0.7	0.765

^†^ PTH: parathyroid hormone, ^‡^ VAS: Visual analogue scale for pain, 0 to 10.

**Table 3:** Pathological findings.

Variable	BABA (n = 13)	SPRA (n = 11)	*p*-Value
Tumor size (cm)	1.4 ± 1.1	1.5 ± 0.4	0.943
Extrathyroidal extension *			
Absent	7	6	1.000
Present	6	5	
Multiplicity			
Single	6	5	1.000
Multiple	7	6	
Tumor margin			
Negative	11	10	1.000
Positive	2	1	
Lymphatic invasion			
Negative	3	3	0.232
Indeterminate	3	0	
Positive	7	8	
Microvascular invasion			
Negative	11	11	0.397
Indeterminate	1	0	
Positive	1	0	
Numbers of metastatic lymph nodes	6.5 ± 4.1	7.6 ± 5.6	0.572
Total retrieved lymph nodes	25.9 ± 10.7	25.3 ± 9.3	0.875
Level 2 lymph nodes	6.6 ± 3.7	6.5 ± 2.9	0.951
Level 3 lymph nodes	5.3 ± 3.3	5.6 ± 3.4	0.811
Level 4 lymph nodes	5.5 ± 3.0	4.7 ± 2.8	0.452
Level 5 lymph nodes	2.0 ± 3.3	2.1 ± 2.0	0.935
Level 6 lymph nodes	6.5 ± 5.3	6.5 ± 5.4	0.970
*BRAF*^V600E^ mutation			
Negative	4	2	0.649
Positive	9	9	

* Including microscopic and gross extrathyroidal extension.

**Table 4:** Surgical completeness in total thyroidectomy and completion thyroidectomy patients.

Variable	BABA (n = 13)	SPRA (n = 11)	*p*-Value
Postoperative 3 months Tg * (mean ± SD, ng/mL)	0.7 ± 1.4	0.3 ± 0.2	0.312
1st RAI ^†^ dose (mean ± SD, mCi)	127.3 ± 26.1	125.0 ± 37.8	0.886
TSH level before RAI (mean ± SD, uIU/mL)	105.9 ± 21.0	137.5 ± 76.2	0.286
Stimulated Tg level before RAI (mean ± SD, ng/mL)	3.5 ± 4.3	5.6 ± 8.6	0.530

* Tg: thyroglobulin, ^†^ RAI: radioactive iodine.

## Data Availability

The data that support these findings are available upon request from the corresponding author.

## Abbreviations

The following abbreviations are used in this manuscript:

SP	Single Port
BABA	Bilateral Axiilary Breast Approach
SPRA	Single Port Robotic Areolar
MRND	Modified Radical Neck Dissection
SCM	Sternocleidomastoid muscle
IJV	Internal jugular vein
CCA	Commom carotid artery
PTH	Parathyroid hormone
VAS	Visual analogue scale
